# Isolated renal mucormycosis presenting with bilateral renal artery thrombosis: a case report

**DOI:** 10.1186/s12301-021-00193-3

**Published:** 2021-06-30

**Authors:** P. S. Saneesh, Raghav Yelamanchi, Shalini Pilllai

**Affiliations:** 1 Deapartment of Radiology, Aster MIMS, Kannur, Kerala India; 2 Department of Surgery, Ward 17, Atal Bihari Vajpayee Institute of Medical Sciences and Dr. Ram Manohar Lohia Hospital, New Delhi, 110001 India; 3 Department of Pathology, Carithas Hospital, Kottayam, Kerala India

**Keywords:** Mucormycosis, Acute kidney injury, Septic thrombus, Bilateral renal artery thrombosis, Sepsis, Case report

## Abstract

**Background:**

Mucormycosis is a rare infection caused by the fungus belonging to the order *Mucorales*. Mucormycosis predominantly affects immunocompromised individuals such as people with acquired immunodeficiency syndrome, blood malignancies, organ transplant, etc. Involvement of the kidneys usually occurs as a result of disseminated mucormycosis. We report a very rare case of isolated renal mucormycosis in an immunocompetant individual without any prior comorbidities who had an unusual presentation of mucormycosis.

**Case presentation:**

A 17-year-old male student had presented to our emergency department with complaints of bilateral loin pain and fever for 10 days. There was no urine output for 2 days. Patient was in sepsis with acute kidney injury. A Doppler ultrasound of the abdomen revealed bilateral enlarged kidneys with absent blood flow in the renal vasculature. Dialysis was done, and patient was started on intravenous antibiotics. Patient was investigated for thrombophilia, the test results of which were normal. Sickle cell test was negative. Immunodeficiency screening was negative. Contrast-enhanced computed tomography revealed bilateral enlarged kidneys with bilateral renal artery thrombosis and mild ascitis. CT-guided renal biopsy was performed in the same sitting which revealed fungal hyphae in the background of necrotic glomeruli. Patient was started on liposomal amphotericin B with renal replacement therapy. However, patient deteriorated and succumbed to sepsis on the 4th day of admission.

**Conclusion:**

Isolated renal mucormycosis with bilateral renal artery thrombosis is a very rare clinical scenario with high mortality. One must have a high degree of suspicion to diagnose renal mucormycosis at an early stage.

## Background

Mucormycosis is a rare infection caused by the fungus belonging to the order *Mucorales* [1]. This fungus is distributed all around and is present in the soil, decaying organic matter, manure, poorly maintained indoor ventilation devices and carpets [2]. Spores are the infective forms of the organism and spread predominantly through inhalation. Mucormycosis predominantly affects immunocompromised individuals such as people with acquired immunodeficiency syndrome, blood malignancies, organ transplant, etc. [3]. Rhinocerebral and pulmonary mucormycosis are the most common manifestation of the disease. Involvement of the kidneys usually occurs as a result of disseminated mucormycosis. The present pandemic of the coronavirus disease 2019 (COVID-19) has increased the incidence of mucormycosis. Apart from the increased number of cases, several rare occurrences of mucormycosis in unusual locations are being reported during this pandemic in India. This is attributed to the excessive use of steroids and immunosuppressive medication in patients of COVID-19. Treatment options are limited for mucormycosis, and the mortality is very high. Hence, one must be vigilant to diagnose the disease at an early stage. We report a very rare case of isolated renal mucormycosis in an immunocompetant individual without any prior comorbidities or COVID-19 infection history who had an unusual presentation of mucormycosis.

## Case presentation

A 17-year-old male student has presented to our emergency with complaints of bilateral loin pain and fever for 10 days. There was no urine output for the last 2 days. There was no significant past, personal, family or genetic history. There was no history of COVID-19 infection or any related symptoms. On examination, patient was sick-looking, pulse was feeble and tachycardic. Blood pressure was 90/60 mm of Hg. On abdominal examination bilateral renal angle tenderness was observed. Patient was resuscitated and investigated further. A Doppler ultrasound of the abdomen revealed bilateral enlarged kidneys with absent blood flow in the renal vasculature. His hemoglobin was 9.1gm/dl, and leukocyte count was 36,000cells/mm^3^. The serum creatinine was 6.5 gm/dl, and serum potassium was 5.8 mEq/L. Arterial blood gas analysis showed metabolic acidosis, for which sodium bicarbonate correction was given. After resuscitation and inotropic support a provisional diagnosis of bilateral acute pyelonephritis with acute kidney injury and renal artery thrombosis was made. Blood cultures were sent. Due to anuria, urine specimen was not available. Dialysis was done, and patient was started on intravenous ceftriaxone and clindamycin antibiotics. Patient was investigated for thrombophilia, and the results of which were normal. Sickle cell test was negative. Immunodeficiency screening was negative. Blood culture was sterile after 72 h of incubation. Chest X-ray was normal.

**Fig. 1:**
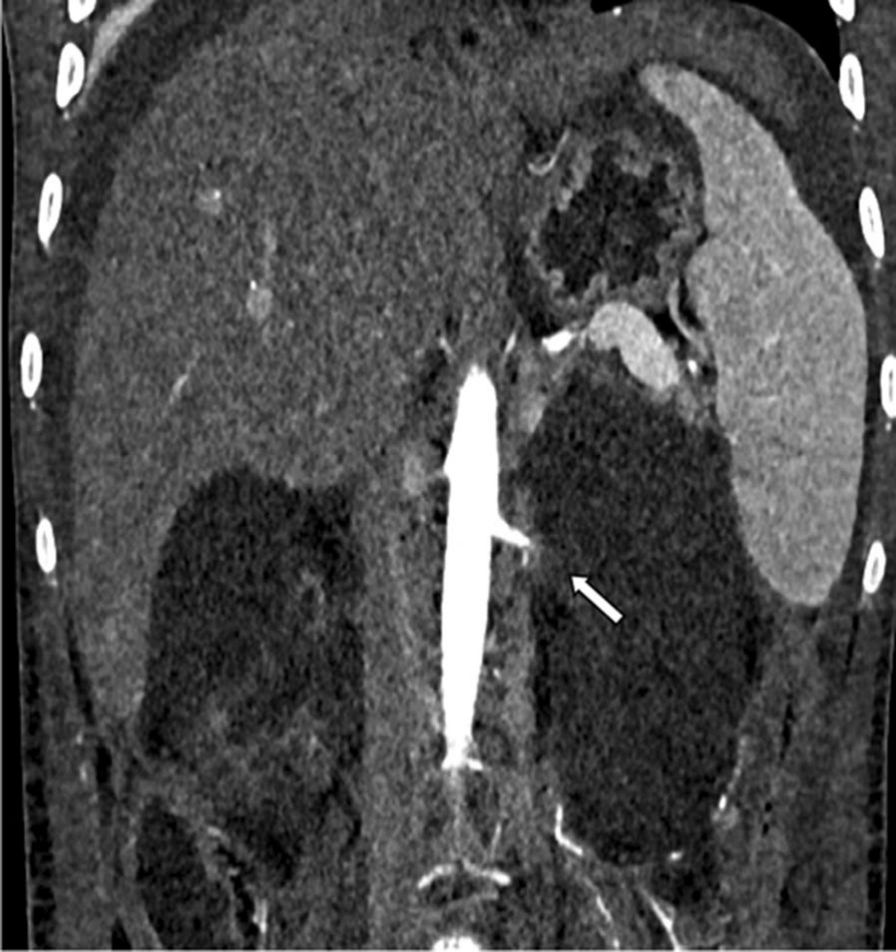
CECT abdomen axial section in arterial phase

**Fig. 2:**
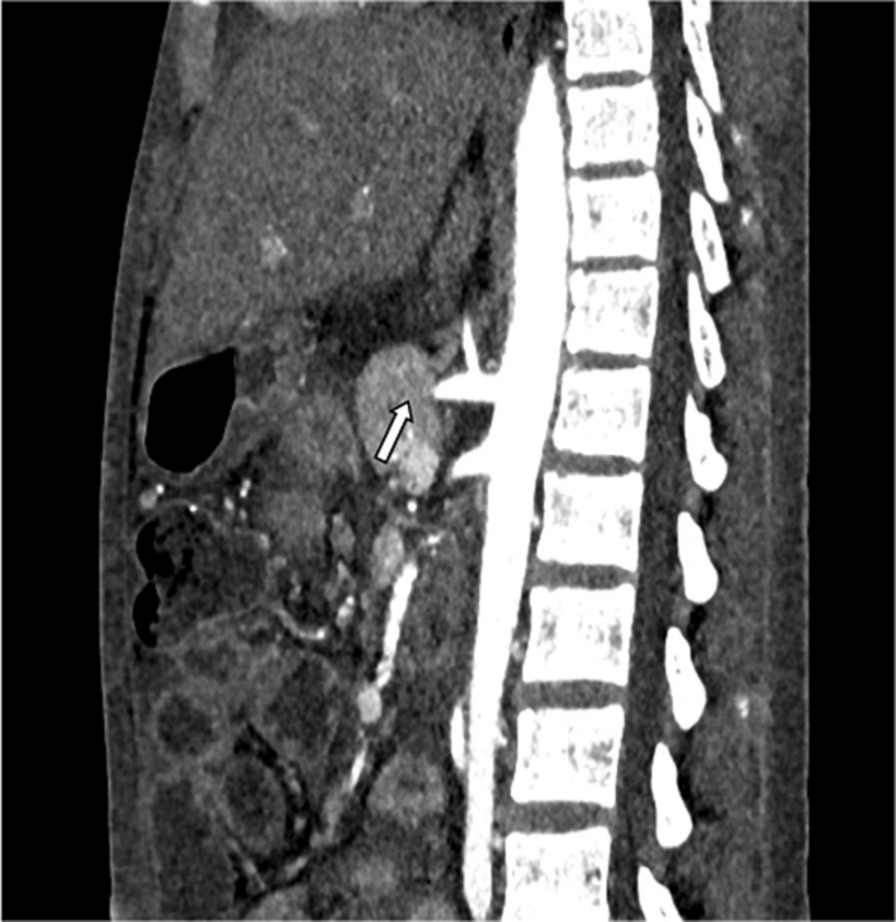
Reformatted coronal arterial phase image

**Fig. 3:**
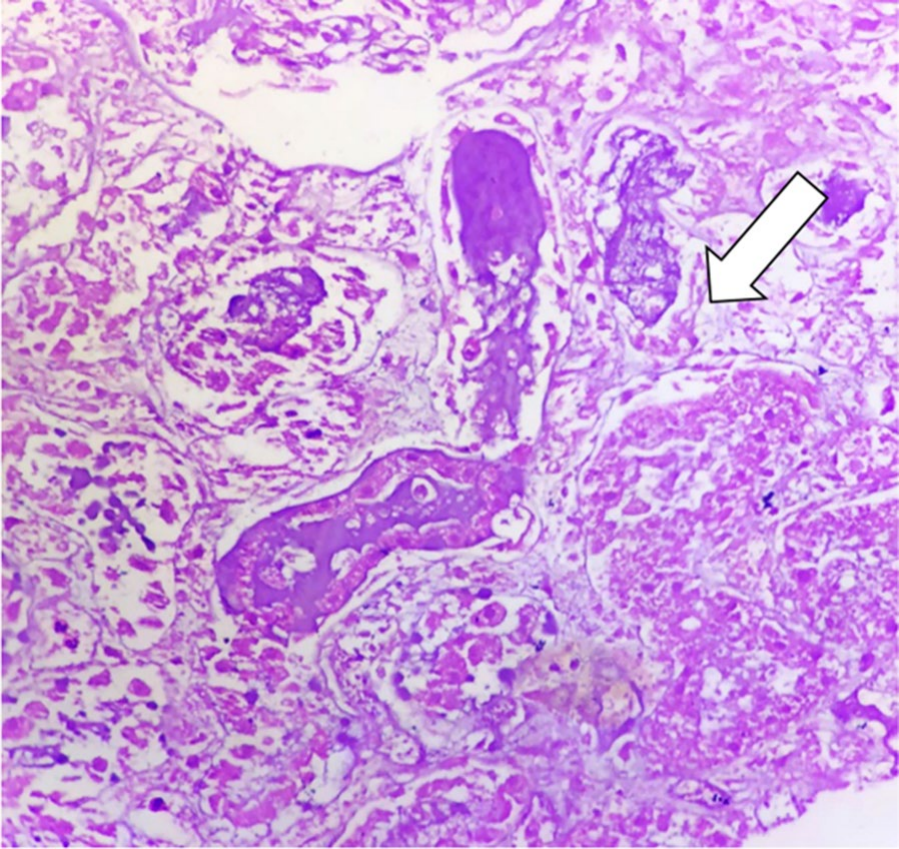
Histopathology image showing necrosis of the glomeruli with fungal hyphae H and E (400×)

The patient did not improve clinically, so it was decided to further investigate the etiology on the third day of admission. Under high-risk consent and peri-procedural dialysis support, contrast-enhanced computed tomography (CECT) was performed. CECT revealed bilateral enlarged kidneys with bilateral renal artery thrombosis with global renal infarcts and mild ascitis (Figs. 1 and 2). CT-guided renal biopsy was performed in the same sitting which revealed fungal hyphae in the background of necrotic glomeruli (Fig. 3). Bronchoalveolar lavage specimen did not reveal any fungal hyphae. Patient was started on liposomal amphotericin B with renal replacement therapy. However, patient deteriorated and succumbed to sepsis on the 4^th^ day of admission.

## Discussion

The above case is very unique in the sense that the mucormycosis occurred in an apparently healthy individual. Only few case reports of mucormycosis in healthy individuals have been reported [4]. Mucormycosis affecting the kidney in isolation is reported mostly in patients who have undergone renal transplant or immunosuppression. A case series of 10 cases of isolated renal mucormycosis in healthy individuals was reported from India by Bhadauria D et al. [5]. As in the present case, most of the patients in the above case series were young males and presented with fever, loin pain and sepsis and were initially misdiagnosed as having acute pyelonephritis [5]. The median duration of illness before the diagnosis of mucormycosis in the case series by Devana SK et al. was 23 days [6]. Hence, in all cases of acute pyelonephritis fungal etiology should be strongly considered, and urine specimens and blood cultures for fungal hyphae should be strongly considered to make an early diagnosis [5].

The presentation of renal mucormycosis in transplant patients is no different than normal individuals with fever and loin pain being the predominant features. Both the transplanted and in-situ kidney can be affected. However, one should note that the most common presentation of mucormycosis in patients of renal transplant is pulmonary and rhino-cerebral mucormycosis. In one of the largest case series of renal mucormycosis in immunocompetant hosts by Devana S.K. et al., four out of 15 patients had bilateral involvement [6]. Other rare presentations of renal mucormycosis include bilateral hydronephrosis [7]. Even in a case of disseminated mucormycosis involvement of kidney is not a common finding [8].

However, only one case of mucormycosis presenting with bilateral renal artery thrombosis in an immunocompetent individual has been reported so far [9]. This case also presented in similar way as in the present case report with fever, loin and anuria [9]. Mucormycosis is known for angioinvasion which may be the reason for the bilateral renal artery thrombosis in the present case report [3]. It is by the same mechanism by which the organism spreads in the blood stream.

In most of the case reports and case series, renal mucormycosis was diagnosed in clinically diagnosed cases of acute pyelonephritis who did not respond to treatment with antibiotics, which prompted further imaging and investigations [5, 6]. One of the consistent features of renal imaging for mucormyosis was kidney enlargement with infarctions either focal or diffuse [5, 6, 10]. Other features were perinephric fat stranding and thickened Gerota’s fascia [6]. Apart from an ultrasound, CECT was used for the diagnosis in most of the cases in order to confirm the findings of the ultrasound as well as to rule out other intra-abdominal causes of sepsis [5, 6]. Biopsy was the only reliable option for the diagnosis in the above case. The blood tests for fungal cultures take several weeks. In most of the case reports, urine specimens were used for the diagnosis of the fungal hyphae [5, 6]. Due to anuria, urine could not be used as a specimen in the present case report. Early diagnosis using polymerase chain reaction is a reliable method but it is not available at all centers [11].

The drugs effective against *Mucorales* include amphotericin B, posaconazole and isavuconazole [12]. Liposomal amphotericin B is the most commonly used drug as it has lesser side-effects and better tolerability than the conventional preparation. In most of the case series, amphotericin B alone or in combination with posaconazole was used. However, the response of the patients to these anti-fungals was not encouraging as is evident in the case reports, in which more than half of the patients required nephrectomy or interventions in the form of percutaneous nephrostomy drainage (PCN) [5, 6, 9]. In the case series by Devana S.K. et al., the decision for nephrectomy was taken after assessing the response 24 h after administering the anti-fungals [6]. The standard dose for amphotericin B is 1 mg/kg/day which should be halved if there is renal impairment. There are few studies which explored the role of high dose (10 mg/kg/day) of amphotericin B with some benefit [13].

Cases like in the present case report, in which the vasculature to the organ is blocked, the drug may not reach the target site compromising its efficacy. The options were very limited for the treatment of the above case. Aggressive anti-fungal therapy with renal replacement therapy may be of initial help to tide over the sepsis. The chance for recanalization of the renal arteries is very meager. A definitive option if the patient recovered from acute crisis would have been bilateral nephrectomy followed by renal transplantation. The mortality of renal mucormycosis was very high (more than 40%) in the reported case series with the mortality rate for bilateral disease reaching 100% [5, 6].

Patients who have recovered from acute crisis were continued on amphotericin B in various case reports. and some case reports also reported the use of maintenance posaconalzole for 3 months after recovery. In cases where the infection was predominantly localized in the pelvi-calyceal system, PCN infusion of amphotericin B was also tried.

## Conclusion

Isolated renal mucormycosis with bilateral renal artery thrombosis is a very rare clinical scenario with high mortality. One must have high degree of suspicion to diagnose renal mucormycosis at an early stage. Reporting of such rare presentations and clinical scenarios is necessary to share the experience of dealing with these cases so as to formulate an optimum diagnostic and treatment algorithm.

## Data Availability

Available on personal request from authors.

## Abbreviations

CECT: Contrast enhanced computerized tomography
CT: Computerized tomography
